# The Computed Microwave Spectrum of the Protonated Fullerene C_60_H^+^

**DOI:** 10.1021/acsearthspacechem.6c00110

**Published:** 2026-08-05

**Authors:** Laszlo Nemes, Jos Oomens, Vincent J. Esposito, Vincent Boudon, Alexander G. G. M. Tielens

**Affiliations:** † HUN-REN Research Center for Natural Sciences, Institute of Materials and Environmental Chemistry, 1117 Budapest, Magyar Tudósok Körutja 2, Hungary; ‡ 98810 HFML-FELIX, Toernooiveld 7, 6525 ED Nijmegen, The Netherlands; § Institute for Molecules and Materials, Radboud University, Heyendaalseweg 135, 6525 AJ Nijmegen, The Netherlands; ∥ Schmid College of Science and Technology, 6226 Chapman University, 1 University Drive, Orange, California 92866, United States; ⊥ Laboratoire Interdisciplinaire Carnot de Bourgogne, UMR 6303 CNRS-Université Bourgogne Europe, 9 Avenue Alain Savary, BP 47 870, F-21078 DIJON Cedex, France; # Astronomy Department, University of Maryland, College Park, College Park, Maryland 20742, United States

**Keywords:** protonated fullerene C_60_, rotational spectra, rotational simulations, quantum chemical calculations, anharmonic vibrations, radio astronomy

## Abstract

The largest known molecule in the interstellar medium of galaxies, C_60_, has been detected in its neutral and cationic form through its vibrational, UV-driven fluorescence emission spectrum and its electronic absorption spectrum, respectively. The detection of several polycyclic aromatic hydrocarbon molecules through their pure rotation spectrum in cold, dense, molecular cloud cores suggests that C_60_ might be present in these environments as well. The low flux of UV pumping photons in molecular cloud cores and the absence of suitably bright background stars, make detection of C_60_ and its cation through the commonly used methods impractical. As C_60_ has no permanent dipole moment, its pure rotational transitions are forbidden, and its presence must be inferred from the rotational transitions of C_60_ derivatives with permanent dipole moments. Here, we present a study of the predicted rotational spectrum of protonated C_60_ that has a sizable permanent dipole moment. Protonation of C_60_ reduces the icosahedral symmetry to C_s_ and results in a dipole moment of about 3.8 D. The resulting C_60_H^+^ is a closed shell system with no electron spin. The ground electronic symmetry is A’. The goal of the present calculations is to simulate rotational spectra in radio astronomy frequency ranges. The simulations are based on geometry and electric dipole moment values from harmonic and anharmonic density functional theory (DFT) calculations at the B3LYP level. Using the PGOPHER spectral simulation program, the rotational structure of the spectra at excitation temperatures of 5 and 10 K were computed to guide future laboratory studies and facilitate radio astronomy searches for protonated C_60_ in cold dark molecular clouds.

## Graphical abstract

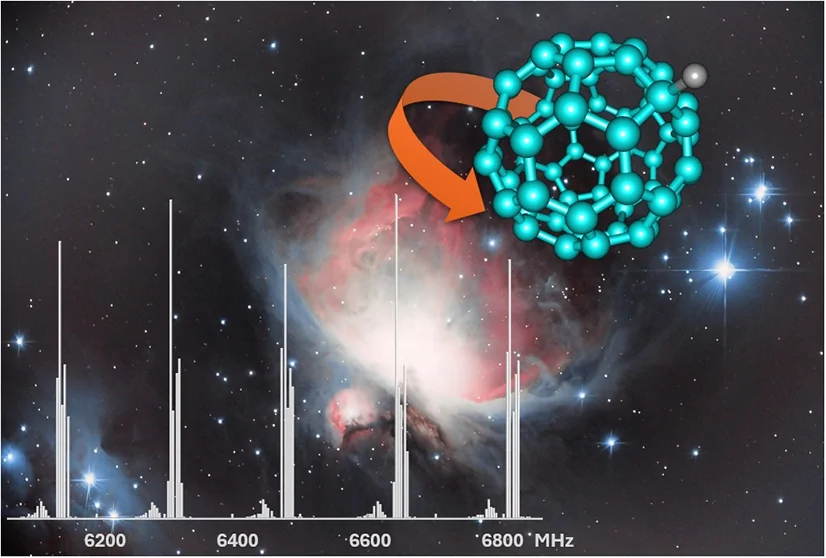

## 1 Introduction

Following the discovery of the fullerenes^1^ in 1985, the macroscopic synthesis of C_60_ by Krätschmer et al.^2^ and the Nobel prize in Chemistry given to Harold Walter Kroto, Robert Curl and Richard Smalley in 1996, interest in fullerenes quickly increased and resulted in an almost exponential growth of the number of publications. Originally, Kroto was interested in the cosmic occurrence of linear cyanopolyynes, which led to his basic interest in the presence of fullerenes in the interstellar medium. The fundamental question was whether some of the so-called diffuse interstellar bands (DIBs) could also be due to fullerenes. Historical clarification on DIBs started with the first observations by Annie Jump Cannon and E.C. Pickering.^3^ The research on DIBs continued in 1922 by the observation of sharp spectral absorption features in astronomical spectra by Mary Lea Heger.^4^

DIBs occur generally as broad diffuse bands in the visible and infrared spectral ranges in the spectra of background stars, where their light traverses relatively low-density material.^5^ It was assumed already early on that the DIBs were due to large carbon bearing molecules. To date, there are some 500 known DIBs, but the difficulty in assigning them to specific molecules remains and is illustrated by the fact that so far there is only one definite molecular assignment: the fullerene radical cation C_60_^+^ having two electronic bands observed in diffuse clouds. After preliminary identification of the C_60_^+^ DIBs by Foing and Ehrenfreund^6^ in 1994, following Fulara et al.,^7^ these two DIBs were finally identified based on laboratory gas-phase spectra obtained through sophisticated laser spectroscopy methods in the Maier group in 2015.^8^ Three weaker DIBs have now also been assigned to C_60_^+^.^9^

The infrared spectra of many astronomical objects are dominated by broad emission bands due to vibrational relaxation of Polycyclic Aromatic Hydrocarbon (PAH) molecules.^10^ These emission features are associated with regions illuminated by strong ultraviolet radiation fields −so-called photodissociation regions^11^ −that pump these molecules electronically. The ensuing vibrational cascade produces bright infrared emission that can be detected using e.g. space borne instruments.^10^ In 2010, the vibrational bands of neutral C_60_ and C_70_ were identified in the IR spectrum of the Tc-1 planetary nebula.^12^ (See also the interview paper with Jan Cami.)^13^ A number of further cosmic C_60_ detections followed in other sources by several authors, e.g. also using the Spitzer Space Telescope.^14^

It has also been suggested that fullerenes and their derivatives could be responsible for the 2175 Å feature in the interstellar extinction curve as well as the 4430 Å DIB.^15,16^ To explain the observed strength of these two DIB features, the implied abundance of fullerenes in the diffuse ISM has to be ∼10^–7^ per H atom. This is a factor 30 larger than the abundance of C_60_^+^ (3×10^–9^ per H nuclei) nuclei derived from the far-red DIBs^17,18^ or the abundance of C_60_ derived from IR observations of the reflection nebula, NGC 7023 close to the exciting star (10^–9^ per H nuclei).^19^ Much higher abundances have been derived from the IR observations of circumstellar media; i.e., the derived abundance of C_60_ in the planetary nebula, Tc1, is ∼10^–7^ relative to H nuclei^12^ and a similarly high abundance was derived for C_60_^+^ in the protoplanetary nebula, IRAS 01005 + 7910.^20^ The abundance of C_60_ and its derivatives in dense molecular cloud cores is unknown but it is quite conceivable that the abundance of this very stable molecule is comparable to or even exceeds the inferred abundance of coronene (10^–9^ per H atom) in TMC-1.^21^ As these studies imply, C_60_ and its derivatives could be very abundant in the interstellar medium.

These studies on the presence of C_60_ in space are limited to regions that lie in front of bright background stars −thus allowing electronic absorption spectroscopy −or that are irradiated by strong ultraviolet radiations fields −producing bright infrared emission. However, in recent years, rotational spectroscopy in the GHz range has uncovered unexpectedly high abundances of small aromatic species, in particular indene and derivatives of naphthalene, pyrene and coronene, in dark molecular cloud cores such as TMC-1, regions that cannot be probed using visible absorption or IR emission spectroscopy due to the dimness of background stars and the absence of UV radiation.^21–23^ These aromatic species point toward the importance of gas-phase chemistry routes for the formation of aromatic species inside cold dense molecular cloud cores. It is conceivable that these routes may also form species as large as C_60_. C_60_ and C_60_^+^ have no permanent dipole moment and cannot be detected in dark cloud cores through their rotational emission. The best method to infer their presence in these environments is through the rotational emission of C_60_ derivatives with permanent dipole moments, such as protonated C_60_.

In general, the inventory of cosmic fullerenes and PAH molecules is not yet well-known and the chemical processes that lead to their formation and destruction are still under investigation. Electronic spectra would be useful to find C_60_H^+^ in interstellar spectra. The gas-phase visible absorption spectrum of protonated C_60_ has been studied using helium nanodroplet techniques and the spectrum reveals a broad absorption band without a counterpart in the DIB spectrum.^24^ The spectrum of fully hydrogenated C_60_ (C_60_H_36_) has been studied in a hexane solution and it has been suggested that this species is the carrier of the well-known 2175 Å bump.^25^ However, solvation in hexane artificially broadens absorption bands and this identification is not unambiguous. In infrared spectroscopy, molecules may have overlapping IR spectra, challenging their individual detection, as is for instance the case for PAH molecules. The infrared spectrum of protonated C_60_ is an exception as C_60_H^+^ will show a CH stretch band in the range 2800–2850 cm^–1^ (3.5–3.57 μm), some 200 cm^–1^ downward of the aromatic CH stretch and some 100 cm^–1^ downward of the characteristic CH stretch for H bonded to sp^3^ C.^26^ No such band is present in the JWST spectrum of the Orion Bar, implying a low abundance. However, this may reflect the low binding energy of H and H^+^ to C_60_ (2.1 and 2.6 eV, respectively) and the ease of photo fragmentation in the UV-rich environment of a photodissociation region.^18,26^ The environments of diffuse clouds and dense molecular cloud cores have much lower UV field intensities and the abundance of hydrogenated C_60_ can be much higher, but, by the same token, the UV fluorescence pump is much less effective and their infrared signature will be difficult to detect. In microwave spectroscopy, spectral lines rarely overlap among different molecules as the rotational frequencies are sensitive functions of the rotational constants. Thus, microwave methods provide powerful tools to identify large carbon molecules in space. However, only polar molecules possess rotational spectra and therefore, the basic fullerenes C_60_ and C_70_ cannot be detected by radio astronomy. Note that most PAH molecules are also apolar or possess only small permanent dipole moments. Ionized fullerenes are usually polar and may be accessible by radio methods. An important counter example is C_60_^+^, in which ionization leads to a Jahn–Teller effect that reduces the icosahedral symmetry to D_5d_,^27^ which is an apolar symmetry group. Thus, C_60_^+^ cannot be detected by radio methods. The Jahn–Teller effect in C_70_^+^ similarly reduces the D_5h_ symmetry of the neutral molecule to C_s_ and this ion has a dipole moment of about 1.3 D; its pure rotational spectrum has been addressed previously.^28^ In the present work, we report a computational study of the rotational spectrum of protonated C_60_ (C_60_H^+^) that has a C_s_ geometry and an anharmonically computed dipole moment of about 3.8 D. We argue that it could thus be useful as a proxy to detect C_60_ in various cosmic sources. The present calculations of the rotational spectrum are based on harmonic^29^ and anharmonic^26^ quantum-chemical calculations. Scaled harmonic frequency calculations were used originally for the analysis of the vibrational spectrum of C_60_H^+^ by Palotás et al.,^29^ and anharmonic frequency calculations were required to rationalize the band position of the single C–H stretch mode.^26^ The present study reports computed rotational constants, other electrical polarity quantities and the predicted pure rotational spectrum.

## 2 Quantum-Chemical Methods

The B3LYP functional in combination with the 6–311+G­(d,p) basis set was selected because of its favorable performance in predicting the vibrational spectrum.^29^ In the calculations presented here the symmetry was constrained to C_s_ and we verified that all vibrational frequencies are real, so that the corresponding geometry is a true minimum.

To compute the anharmonic spectrum, the geometry of C_60_H^+^ was optimized in C_s_ symmetry, using the B3LYP density functional in combination with the 6–31G basis set. This smaller basis set was used to compensate for the high computational cost of anharmonic frequency calculations for large molecules. The optimization used tight convergence criteria (energy convergence threshold of 1 × 10^–12^) and employed an integration grid consisting of 200 radial shells and 974 angular points per shell (200,974). Following this, the normal modes, harmonic frequencies, and cubic and quartic force constants were computed at the same level of theory. The higher order force constants were computed using numerical differentiation via finite differences of analytical gradients as implemented in Gaussian 16. From there, second order vibrational perturbation theory was used to compute the anharmonic vibrational frequencies of C_60_H^+^ to obtain the ground-state vibrationally averaged rotational constants as well as the quartic and sextic constants. The size of the computed electric dipole moment is 3.8622 D and the value of the final electronic energy is −2285.928213 hartree. The GAUSSIAN 16 suite of programs^30^ was employed to run geometry optimization and harmonic frequency calculations of C_60_H^+^.

## 3 The Molecular Geometry and Spectral Parameters of C_60_H^+^

Using the ground vibrational state averaged rotational constants and quartic centrifugal constants from anharmonic calculations described in the previous section, the rotational spectra were simulated using the PGOPHER software.^31^ The binding of the proton to a carbon atom of C_60_ by a covalent single chemical bond distorts the geometry from its icosahedral symmetry to C_s_. Figure 1 shows the structure of C_60_H^+^ as obtained from the geometry optimization using the B3LYP/6–311+G­(d,p) functional constrained to C_s_ symmetry, which was used to run harmonic frequency calculations. The proton binds to the carbon by a covalent bond of 1.1035 Å, distorting the original sp^2^ configuration to the tetragonal sp^3^. To understand this transformation, one has to consider that in C_60_, the symmetry axes are not on vertices (on atoms), but in the centers of hexagons and pentagons. Hence, the attachment of a proton to a carbon atom results in a significant symmetry breaking and not simply to a reduction to a symmetric top group. Adding a proton thus leads directly to the asymmetric top symmetry C_s_. Only a single mirror plane is preserved (see Figure 2) and the emerging dipole becomes slightly misaligned with the C–H^+^ bond.

**Figure 1:**
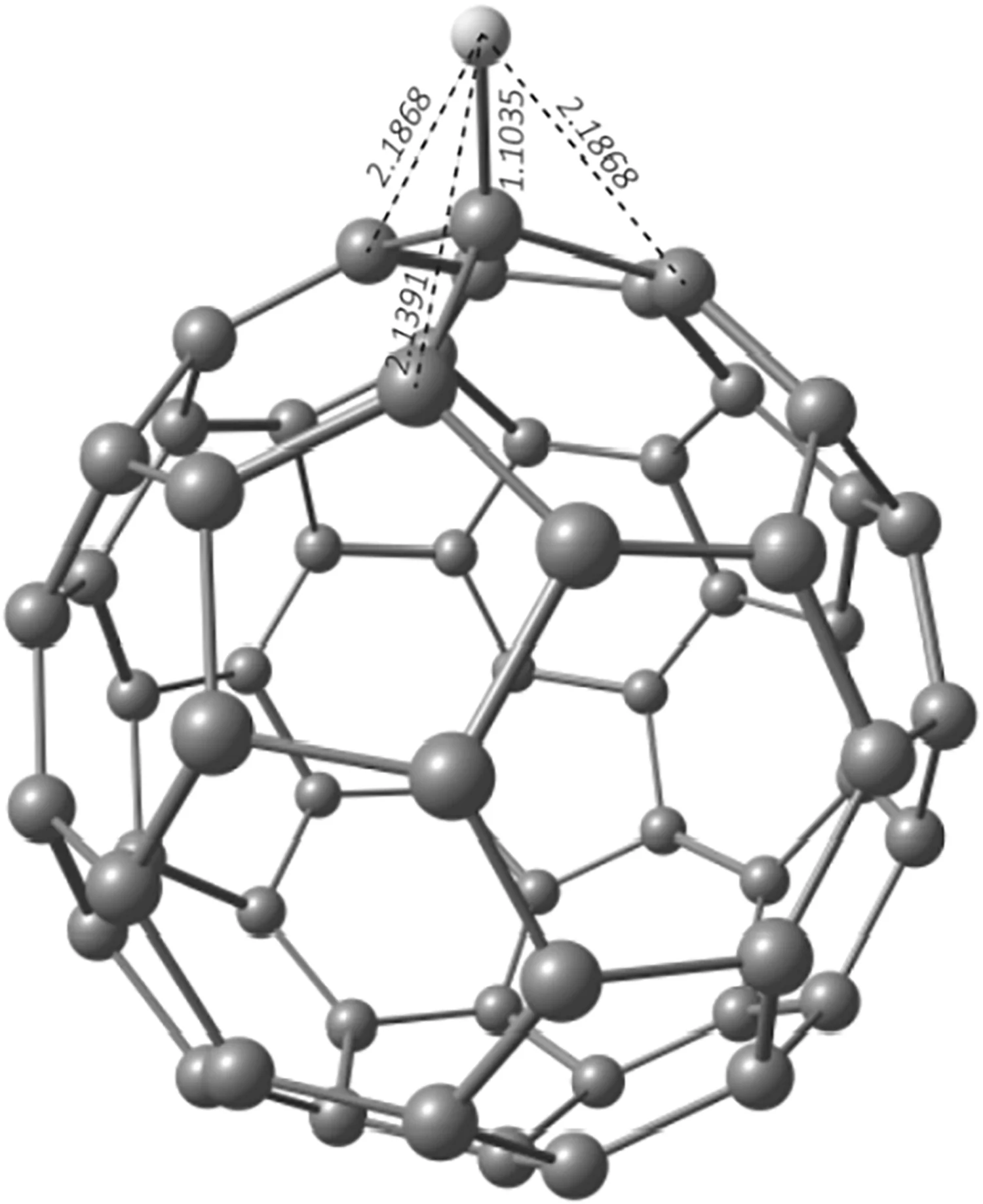
Structure of C_60_H^+^ with the C–H^+^ bond length shown in Angstrom

**Figure 2:**
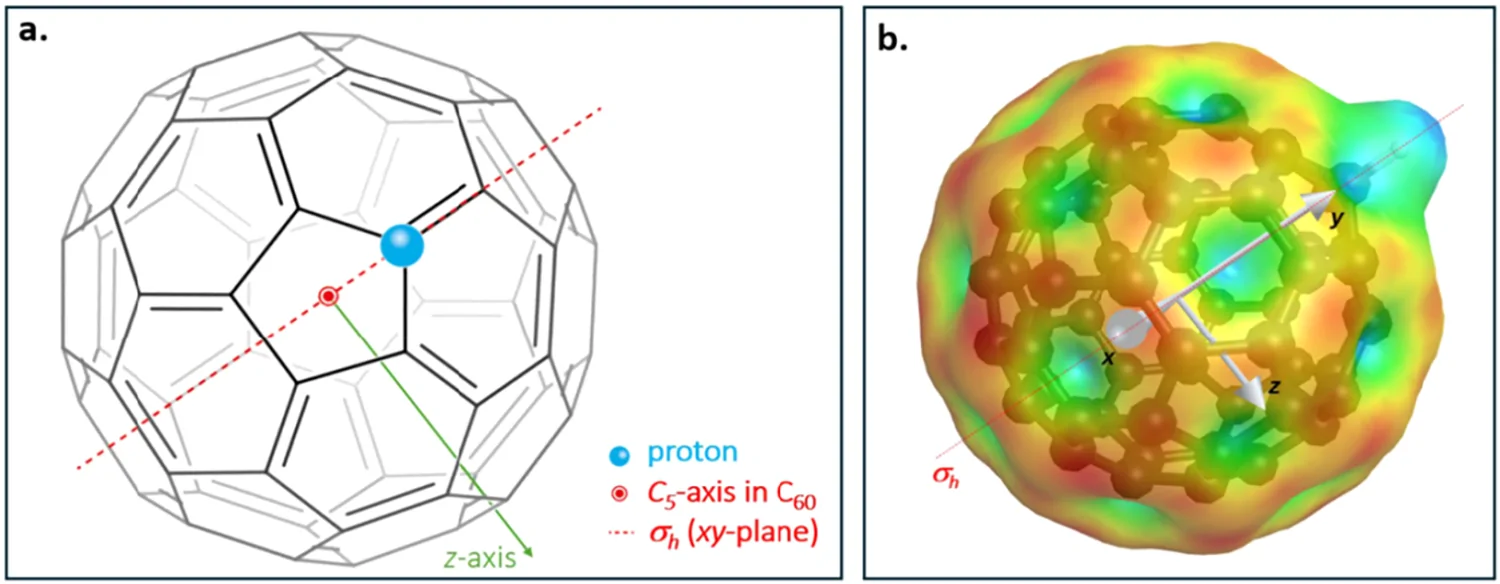
(a.) Sketch of protonated C_60_ showing the molecular mirror plane edge-on, the proton is attached to one of the C atoms. The mirror plane is the only remaining symmetry element. (b.) The electrostatic gradient of the Mulliken charge density of C_60_H^+^ was derived from anharmonic Gaussian 16 calculations and rendered in GaussView. The gradient goes from red to blue, from negative to positive. The size of the dipole is 3.8619 D. The molecule-fixed Cartesian axes x, y and z are indicated; the *z* axis is perpendicular to the xy plane, which contains the C–H^+^ bond and the dipole moment vector components.

The question may arise whether there are structural isomers depending on which carbon carries the proton. However, since all carbon atoms are symmetrically equivalent due to the isolated pentagon rule, all carbon atoms are situated at an apex of two sidewise joined hexagonal rings enclosing a pentagon, so that the proton binding site is immaterial. Singly protonated C_60_ has no distinct structural isomers and there is only one local minimum corresponding to a stable configuration. Calculated energy barriers against migration between equivalent sites range from 1.2 to 3.9 eV, depending on the pathway.^32^ When quantum-mechanical proton hopping (tunneling) occurs between these equivalent sites, spectroscopic features are affected (line broadening or doublet forming) and it is necessary to use a molecular symmetry group containing elements describing proton tunneling motion.^33,34^ However, the experimental IR spectrum of C_60_H^+^ does not show any evidence of abnormal line broadening −in particular, the single C–H+ stretch band is sharp and well-defined^26^ – so that we will not further consider proton tunneling here.

In Figure 2, the molecule-fixed Cartesian axes and the electrostatic potential distribution are shown. The charge density is distributed over the C_60_ body, but near the proton there is a local density maximum. The dipole arises mainly from the separation between the proton charge and the center of mass of the molecular ion. The results of the calculation for the rotational constants and the dipole moments based on the equilibrium **r**_**e**_ structure as obtained from harmonic calculation using the B3LYP/6–311+G­(d,p) level are listed in Table 1.

**Table 1:** Equilibrium Rotational Constants and Space-Fixed Cartesian Dipole Moment Components from Calculations Using the B3LYP/6-311+G­(d,p) level^a^

Parameter	Value
A	83.76 MHz
B	83.47 MHz
C	83.17 MHz
μ_*x*_	1.0771 D
μ_*y*_	3.4012 D
μ_*z*_	0.0000 D
μ	3.5676 D

a The dipole moment components correspond to the field-independent basis used in Gaussian.

For correct rotational intensities, molecule-fixed Cartesian dipole components have to be used, which are usually called the principal moment axis components. The x,y,z axes in Figure 2 are molecule-fixed Cartesian axes.

It is also important to consider that molecules are not rigid even at absolute zero, since the constituting atoms are in zero-point vibrational motion. Therefore, the zero point average geometry is somewhat different from the equilibrium geometry obtained from harmonic vibrational potentials. For small polyatomic molecules, an empirical relationship was obtained between the equilibrium and zero-point average moments of inertia.^35^ It predicts about 0.3% difference between the rotational constants of the r_e_ and r_0_ structures of small molecules. Instead of using this statistical estimate, anharmonic vibrational calculations were actually carried out. Both harmonic and anharmonic runs were performed with C_s_ symmetry imposed (see Section 2.) and the resulting constants are summarized in Table 2. As anharmonic methods are computationally very expensive for such large molecules, the B3LYP/6–31G level was used instead of B3LYP/6–311+G­(d,p), used in the harmonic calculations. The Ray asymmetry parameter, κ = (2B-A-C)/(A-C), is extremely sensitive to the precise values of A, B and C, due to the near-zero value of the denominator for a near spherical top. Hence, independent of the actual value of κ, rather than being a near-symmetric top, C_60_H^+^ is an unusual case of being near-spherical. The Ray asymmetry parameters derived from the computed rotational constants in Table 2 are misleading as they do not suggest that C_60_H^+^ is a nearly spherical top. Therefore, we disregard the Ray asymmetry in the following.

**Table 2:** Computed Rotational Constants (in MHz) in the III_r_ Axis Representation (x,y,z > a,b,c). All Computations Performed With The B3LYP Density Functional

Rotational Constants	r_e_ 6–311+G(d,p)	r_e_ 6–31G	r_0_ 6–31G	r_0_-r_e_ 6–31G difference
A	83.76	82.976	82.433	–0.543
B	83.47	82.632	82.082	–0.550
C	83.17	82.388	81.848	–0.540

While quartic centrifugal distortion constants were used in the simulations, as taken from the anharmonic calculations shown in Table 3, they are so small that they may be disregarded at the J values used in this study and this results in the same effective rotational constants. At higher temperatures (and hence higher J values), however, centrifugal distortions may have to be included in rotational simulations. In the following simulations the rigid rotor model was used.

**Table 3:** Quartic Centrifugal Constants (in MHz) for the III_r_ Asymmetric Top Reduction

Δ_J_	1.7409 ×10^–8^
Δ_K_	–5.1020 × 10^–10^
Δ_JK_	2.4323 × 10^–10^
δ_J_	2.8096 × 10^–11^
δ_K_	–1.2876 × 10^–9^

Table 4 presents the calculated rotational constants obtained using different basis sets to illustrate inherent systematic uncertainties associated with the quantum-chemical calculations. These amount to uncertainties of the order of 0.8 MHz in the rotational constants. In the subsequent spectral calculations, we have adopted the rotational constants of Table 2.

**Table 4:** Comparison of Rotational Constants (in MHz) Obtained Using Different Basis Sets to Illustrate Systematic Uncertainties in the Calculated Rotational Constants^a^

Rotational Constants	r_e_ Def2SVP	r_e_ aug-cc-pVDZ	r_e_ aug-cc-pVTZ	r_e_ 6–311+G(d,p)	r_e_ 6–31G	r_0_ 6–31G	r_0_-r_e_ 6–31G difference
A	83.297	83.356	84.153	83.76	82.976	82.433	–0.543
B	83.009	83.068	83.859	83.47	82.632	82.082	–0.550
C	82.723	82.779	83.554	83.17	82.388	81.848	–0.540

a The Ray asymmetry parameters are left out from this Table.

## 4 Simulated Rotational Spectra of C_60_H^+^

In the present simulations, the rotational constants in the fourth column of Table 2 (6–31G) and ground-state vibrational averaged dipole moment components in the principal axis system were used: μ_a_= 3.6057 D, μ_b_ = −1.383 D and μ_c_ = 0 D. The orientations of the **a**, **b** and **c** principal axes and the direction of the dipole moment are shown in Figure 3. The molecular symmetry plane is indicated in light blue color; axes **a** and **b** are in the symmetry plane and axis **c** is perpendicular to it. The dipole moment direction coincides with the C–H bond, while axis **a** is slightly rotated away.

**Figure 3:**
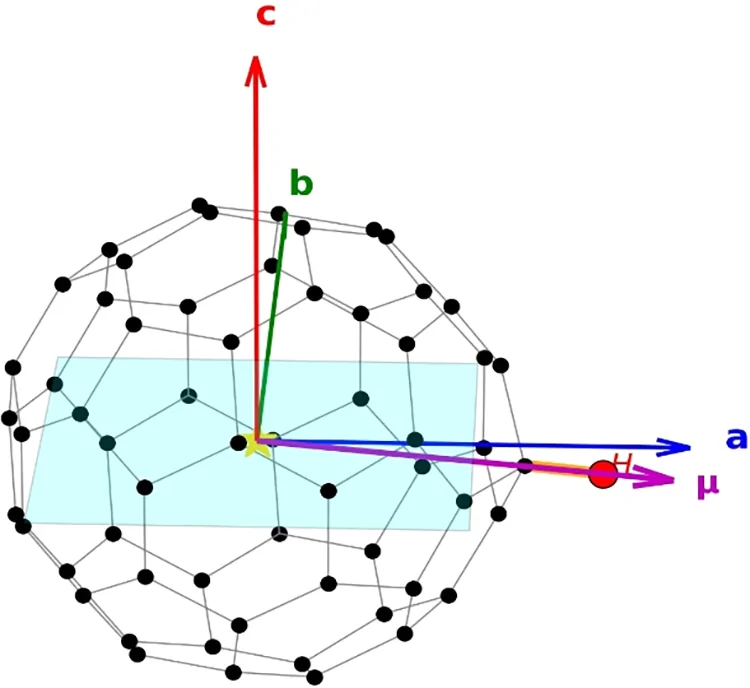
Position of the principal rotational axes in C_60_H^+^.

The positions of the principal rotational axes are not used by the Gaussian anharmonic vibrational calculations, as the positions of the nuclei, dipole components, etc. are referred to a space-fixed static Cartesian system of axes. To obtain the dipole moment components in the molecule-fixed axes, we use the inertial tensor that contains the Eulerian angles, describing the relative position of the two axis systems.^36^ In Table 5, these principal axis dipole moment components in the harmonic and anharmonic equilibrium structures and the anharmonic ground-state vibrational average structure are given (for the corresponding rotational constants see the second to fourth columns in Table 2).

**Table 5:** Principal Axis Dipole Moment Components (in Debye) in the Equilibrium and Ground Vibrational Average State Structures

Computational models	Total	A axis	B axis	C axis
6–311+G(d,p) equilibrium	3.5676	3.2068	1.5636	0
6-31G equilibrium	3.8270	3.5345	–1.4674	0
6-31G ground vibr. aver.	3.8619	3.6057	–1.3830	0

The Einstein A coefficients describe the spontaneous emission rates from the upper rotational levels. The Einstein A coefficient scales with the square of the transition moment in Debye and the cube of the line frequency in MHz units (Figure 4).

**Figure 4:**
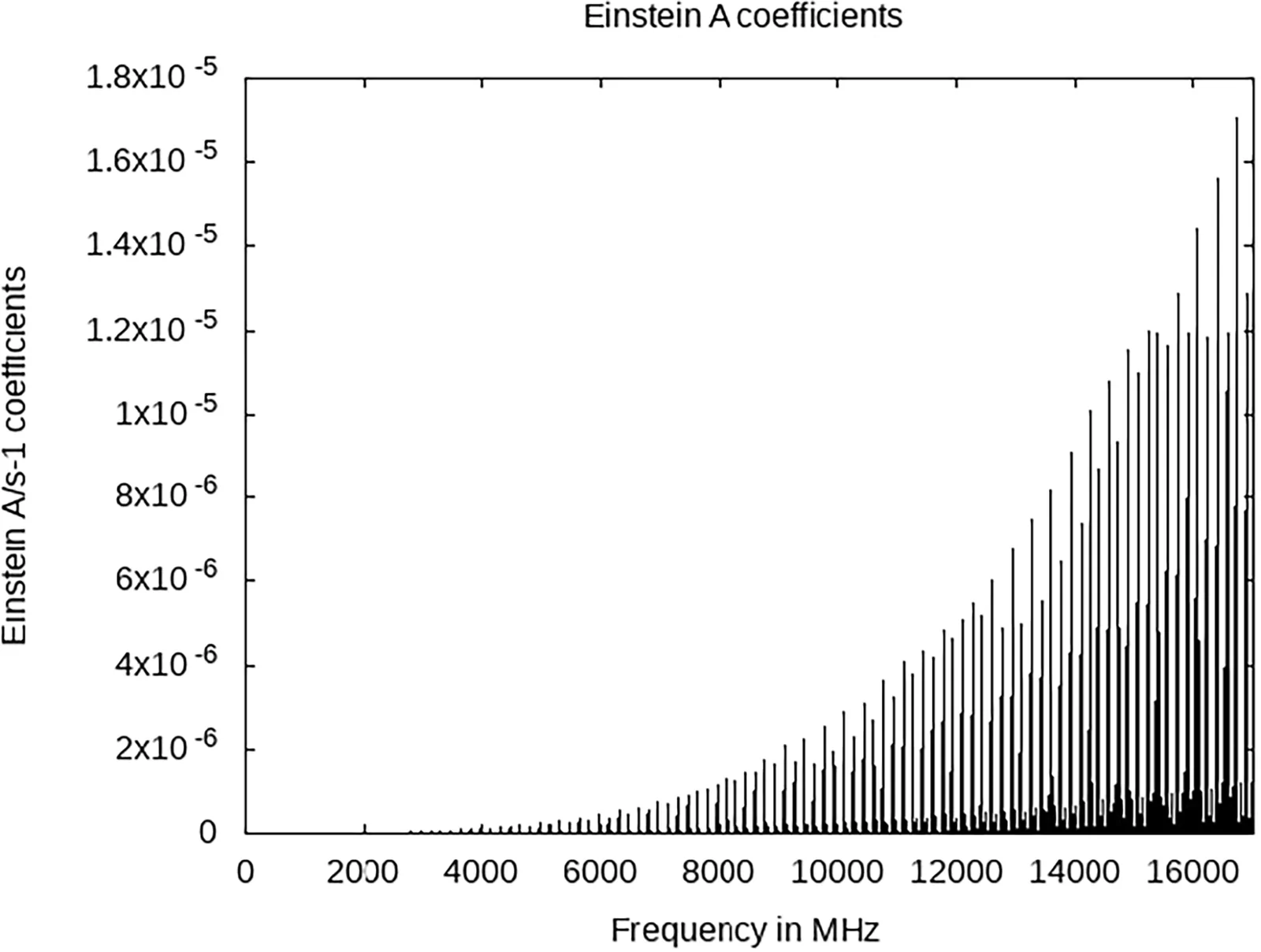
Calculated Einstein A coefficients for C_60_H^+^.

Figures 5–8 show the predicted spectra in normalized intensities, which are the default in PGOPHER and which contain rotational partition functions and Boltzmannian level populations. An excitation temperature of 5 K was chosen in Figure 5 to resemble laboratory molecular beam microwave spectroscopy and dark molecular cloud conditions and applies for thermal equilibrium. The dipole moment components used are those in the last row of Table 5. In the simulations, individual lines were given zero Gaussian and Lorentzian line widths and no Doppler width was used. The dense spectrum contains a large number of spectral lines, merging together at the low resolution of the plot and due to the relatively small number of points used in the simulation. Thus, the intensity fluctuations in Figures 5 and 6 are artifacts and do not correspond to the true rotational structure. The purpose of showing these simulations is to give the frequency range and its shift upon increasing the temperature.

**Figure 5:**
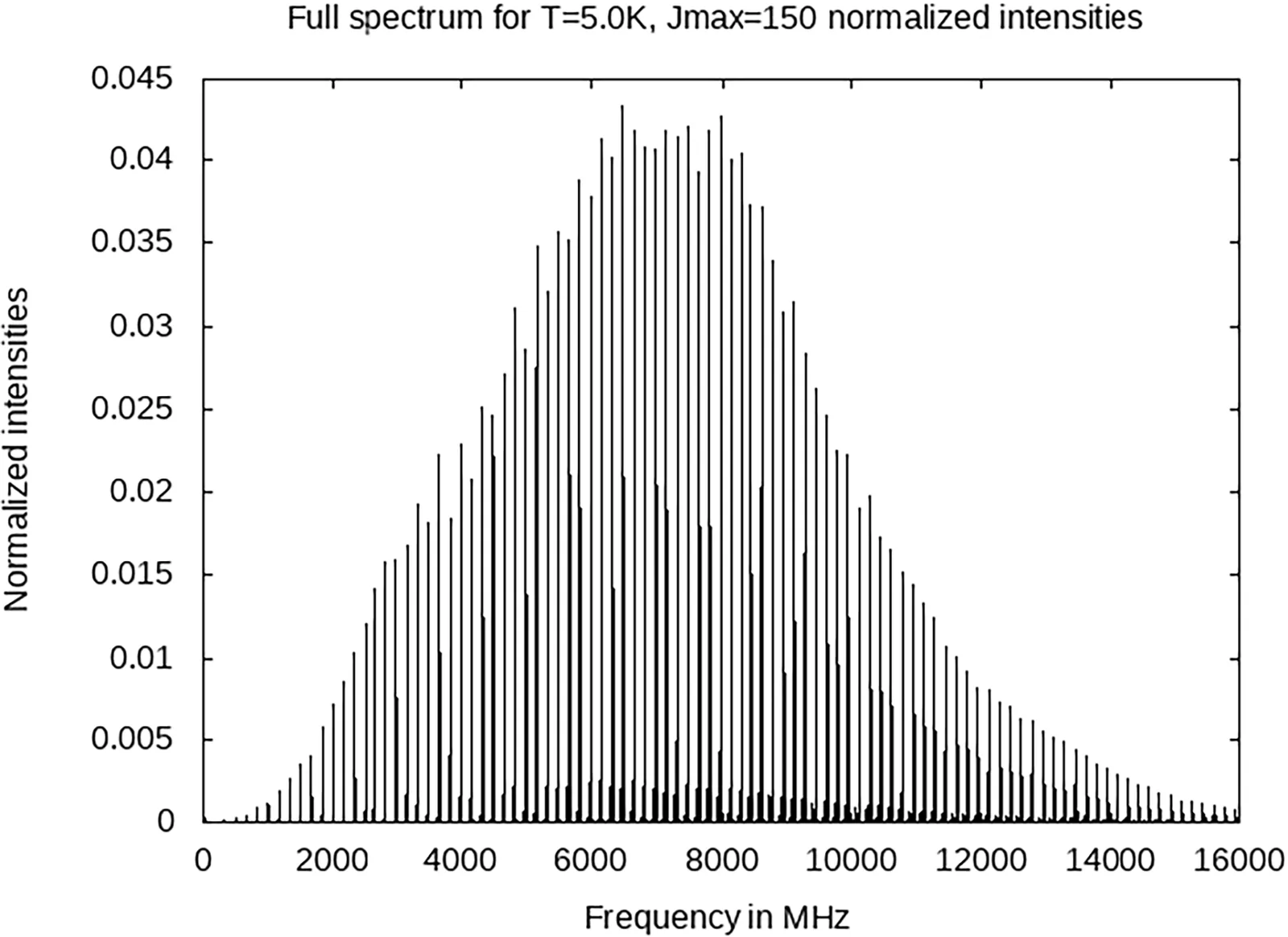
Simulated rotational spectrum of C_60_H^+^ at *T* = 5 K.

**Figure 6:**
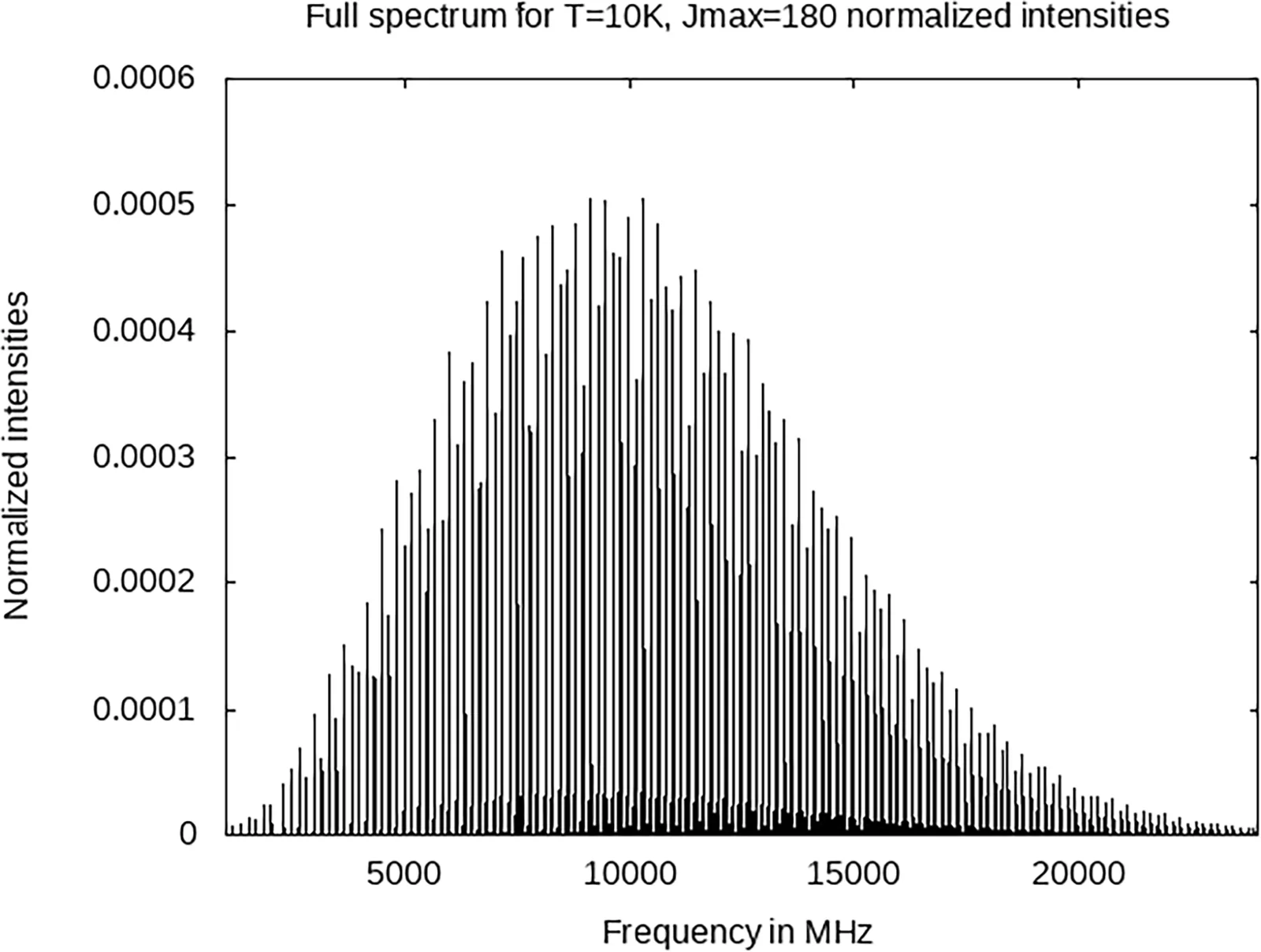
Simulated rotational spectrum at *T* = 10 K.

Figure 8 shows an enlarged portion of the J = 60 sub-branch, which contains only a-type transitions. Most of these form a regular, comb-like arrangement. The separation between the lines in the comb-like part of the spectrum is less than 1 kHz. In asymmetric rotors of the C_s_ point group, the rotational transition symmetry is either A″ or A′; both are dipole-allowed.

In Table 6, representative values of the rotational partition sums for C_60_H^+^ are listed for temperatures of 5, 10, 15, and 20 K, as calculated in the symmetric top approximation. The rotational constants from the second column in Table 2 (6–311+G­(d,p), r_e_) are B_1_ = A = 83.76 MHz, B_2_ = √(B*C) = 83.2934084 MHz, where B_1_ and B_2_ are the rotational constants of the one-dimensional and two-dimensional rotors of the symmetric top.^37^

**Table 6:** Rotational Partition Sums for Four Different Temperatures Calculated Using the Symmetric Top Approximation

Temperature (K)	Q_rot_
5.0	78191
10.0	217155
15.0	372926
20.0	519159

## 5 Discussion

The present work aims at recommending efforts in laboratory microwave spectroscopy and radio astronomy for finding protonated C_60_ in cosmic sources, thus offering detection possibilities in addition to infrared astronomical methods, when laboratory IR spectroscopy^26,29^ and eventually optical spectroscopy of the visible spectrum of C_60_H^+^ will be known. Specifically, rotational spectroscopy offers the opportunity to detect C_60_ derivatives in regions with low UV irradiation, such as dark cloud cores. This can then be used to infer the presence of C_60_ itself and its abundance can be derived indirectly, as is done for the cyanogen derivatives of small PAHs.^21,22^

As emphasized in the Introduction, the low UV density in dark cloud cores inhibits the use of common IR fluorescence detection methods. Likewise, the high dust extinction at visible and far-red wavelengths through such cores hampers the detection of electronic transitions in the spectra of background stars. Chemical studies of neutral and cationic C_60_ have been developed describing the formation of C_60_H^+^ and other hydrogenated fullerene cations.^38,39^ Kroto and Jura hypothesized that the most abundant fullerene analogue in the diffuse interstellar medium is C_60_H^+^.^40^

The radio-astronomical search for polar fullerene forms and derivatives is undoubtedly going to be more difficult than the search for small molecules, because the large rotational partition sums result in a large number of weak lines. This obviously is the case for C_60_H^+^. Table 6 contains a sample of rotational partition sums for very low temperatures. The population of the rotational levels increases quickly at elevated temperatures, so that the spectrum becomes densely filled with lines. At temperatures around 100 K and above, the spectra may even become a weak continuum. The comparison of Figures 5 and 6 demonstrates the shift of the most intense lines from about 7 GHz to about 10 GHz upon temperature increase from 5 to 10 K. Radio astronomical searches for C_60_H^+^ are expected to be the most successful in the coldest cosmic locations. In the cores of dark dense molecular clouds (such as the TMC-1 Taurus molecular cloud), interstellar dust grains in the coldest, densest part of the cloud extinguish visible and infrared radiation, so that molecular detection is only possible by radio astronomy. In photodissociation regions (PDRs), a star irradiates the surrounding neutral gas with far-ultraviolet photons. Rather than collisional equilibrium, in PDRs, the rotational population is set by UV excitation followed by ro-vibrational cascade. The resulting rotational population can be well approximated by a distribution at an effective rotational temperature of *T*_rot_ = *hcv*_IR_/6*k* with *v*_*IR*_ the average vibrational frequency and the factor of 6 accounts approximately for the sum over the K-ladders.^18^ With *v*_IR_ ≈ 600 cm^–1^, we find *T*_rot_ ≈ 150 K. Thus, the rotational excitation and partition function will be large, causing the rotational spectrum to become very weak and unfavorable for detection by radio astronomy.

The question emerges whether the simulations presented in this work are applicable, considering that these assume thermal equilibrium. This question may be answered with the computed Einstein A coefficients in Figure 4. Because of the larger dipole moment and Einstein A coefficients, collisional depopulation of C_60_H^+^ rotational levels should occur faster than for the neutral radical species. This depopulation factor may be estimated by the so-called critical density term *n*_cr_, given by the ratio of the Einstein A coefficient and the collisional de-excitation coefficient, γ

$$n_{\mathrm{cr}}=\frac{\sum_{l<u}A_{u,l}}{{\sum_{l\neq u}\gamma }_{u,l}}$$

where the u,l indices refer to the upper and lower levels, respectively (See chapter 4 in ref 18.). At densities larger than *n*_cr_, thermal equilibrium is established. For ion-neutral interactions, the collisional de-excitation coefficient, γ, is given by the Langevin rate, ≈ 5 × 10^–8^ cm^3^s^–1^ for H_2_.^18^ From Figure 4, we see that the largest Einstein A coefficient is around 1 × 10^–5^ s^–1^ and the critical density is about 200 cm^–3^. Typical densities are about 10,000 cm^–3^, so that collisions are much faster than radiative de-excitation and therefore thermal equilibrium is established. This result supports the thermal equilibrium model for spectral simulations using the PGOPHER software. Thermal equilibrium is involved in the plots displayed in Figures 5–8.

As to the possibility of emission from protonated fullerenes and similar polar carbon molecules contributing to the anomalous microwave emission (AME),^41^ as proposed by Iglesias-Groth,^42^ high temperature rotational simulations are needed but not yet available. The presently used excitation temperature of 5 K is chosen on the basis of the work of Wenzel et al.^21^ on cyanocoronene and it is appropriate for the dark cloud core TMC-1. Nevertheless, following Tielens,^18,43^ we can estimate the contribution of C_60_H^+^ to the AME. The rotational population is a competition of UV excitation followed by vibrational relaxation, collisional deexcitation, and pure rotational, radiative decay. For diffuse clouds, with a hydrogen density of 30 cm^–3^ exposed to the average interstellar ultraviolet radiation field (10^8^ photons cm^–2^ s^–1^), the frequency corresponding to the most probable J is ∼12.5 GHz and the rotational emission is expected to peak at ∼20 GHz. The expected intensity is then

$$4\pi I_{\mathrm{AME}}\left(C_{60}H^{+}\right)\approx 5.3\times 10^{-22}\mathrm{erg}s^{-1}{\left(C_{60}H^{+}\right)}^{-1}$$

The observed intensity of the AME is^18,43^

$$4\pi I_{\mathrm{AME}}\approx 2.4\times 10^{-30}\mathrm{erg}s^{-1}{\left(H-\mathrm{atom}\right)}^{-1}$$

resulting in a potential contribution of C_60_H^+^ to the AME of

$$\frac{I_{AME}\left(C_{60}H^{+}\right)}{I_{AME}}\approx 0.2\frac{X\left(C_{60}H^{+}\right)}{10^{-9}}$$

Hence, given the high dipole moment of C_60_H^+^, its contribution to the AME in the diffuse ISM could be substantial. Inside dense clouds, the absence of UV radiation will lead to a much lower rotational population and the potential contribution of C_60_H^+^ to the AME is small.

Carefully inspecting the values in Table 2, one notices another important aspect of the quantum-chemical calculations performed for C_60_H^+^: that it is essentially an almost spherical top. The DFT-computed values for the rotational constants obtained in B3LYP/6–311+G­(d,p) and B3LYP/6–31G calculations are rather different, so that computing rotational spectra in the harmonic approximation may not be sufficiently accurate for radio astronomical searches. Considering that the J sub-branch separation is given by (B+C), the sub-branch separations are sensitive to the value of the rotational constants. However, we emphasize that in all cases C_60_H^+^ is near-spherical, and, while the details may change, the calculated peculiar spectral behavior of its rotational spectrum is preserved.

In astronomical studies of the rotational emission of interstellar molecules, intensities are generally given in units of K km/s. Using the intensities provided by PGOPHER and taking I_rot_ in units of Watts/molecule, astronomical intensities have been calculated as

$$T\Delta v=\frac{c^{3}}{{8\pi \nu }^{3}k}I_{rot}N\approx 7.8\times 10^{-4}{\left(\frac{10GHz}{\nu }\right)}^{3}\left(\frac{I_{rot}}{10^{-35}\frac{W}{molecule}}\right)\left(\frac{N_{tot}}{10^{14}\frac{molecules}{cm^{2}}}\right)K\;km/s$$

with *N*_tot_ the total number of molecules along the line of sight. The values in Table 7 have been calculated for a kinetic temperature of 5 K and total C_60_H^+^ column densities of 10^13^ and 10^14^ cm^–2^, which is reasonable given the excitation temperature of 5 K and column density of 3×10^12^ cm^–2^ observed for cyanocoronene.^17^ As intensities depend linearly on the adopted column density, the intensities provided in Table 7 are easily extrapolated to other column densities. For comparison, we estimate a 3σ-detection limit of about 10^–3^ K kms^-1^ from the spectra of the rotational transitions of cyanocoronene in this same frequency range.^21^ This sensitivity can be greatly improved by stacking of lines, as for random noise the sensitivity improves as the square root of the number of lines stacked. In addition, special filtering techniques have been developed to improve the detectability of rotational transitions.^22,44^

**Table 7:** Intensities at T = 5 K of Selected Lines Near 9.8 GHz in Units of K km s^-1^ for Adopted Total Column Densities of 10^13^ and 10^14^ cm^–2^

Frequency	Intensity	*E*_u_	*E*_l_	T_mb_*Δv	
MHz	W molecule^-1^	MHz	MHz	K km s^-1^	
				*N* = 10^14^ cm^–2^	*N* = 10^13^ cm^–2^
9822.13	3.92 × 10^–35^	299585.859	289763.7291	3.22 × 10^–3^	3.22 × 10^–4^
9822.87	5.57 × 10^–36^	299629.806	289806.9355	4.57 × 10^–4^	4.57 × 10^–5^
9823.6102	3.84 × 10^–35^	299672.926	289849.3161	3.15 × 10^–3^	3.15 × 10^–4^
9824.3505	3.80 × 10^–35^	299715.216	289890.8657	3.12 × 10^–3^	3.12 × 10^–4^
9825.0913	3.76 × 10^–35^	299756.67	289931.5787	3.09 × 10^–3^	3.09 × 10^–4^
9825.8325	3.72 × 10^–35^	299797.281	289971.4489	3.05 × 10^–3^	3.05 × 10^–4^
9826.5743	3.69 × 10^–35^	299837.044	290010.4692	3.03 × 10^–3^	3.03 × 10^–4^
9827.3171	3.65 × 10^–35^	299875.949	290048.632	2.99 × 10^–3^	2.99 × 10^–4^
9828.061	3.61 × 10^–35^	299913.99	290085.9286	2.96 × 10^–3^	2.96 × 10^–4^
9828.8064	3.58 × 10^–35^	299951.155	290122.349	2.94 × 10^–3^	2.94 × 10^–4^
9829.5536	3.55 × 10^–35^	299987.436	290157.8824	2.91 × 10^–3^	2.91 × 10^–4^
9831.0556	3.48 × 10^–35^	300057.291	290226.2352	2.85 × 10^–3^	2.85 × 10^–4^
9831.8118	3.45 × 10^–35^	300090.835	290259.0236	2.83 × 10^–3^	2.83 × 10^–4^
9832.5725	3.42 × 10^–35^	300123.434	290290.8616	2.80 × 10^–3^	2.80 × 10^–4^
9833.3392	3.40 × 10^–35^	300155.066	290321.7263	2.78 × 10^–3^	2.78 × 10^–4^
9834.1133	3.37 × 10^–35^	300185.704	290351.5905	2.76 × 10^–3^	2.76 × 10^–4^
9834.8971	3.35 × 10^–35^	300215.319	290380.4215	2.74 × 10^–3^	2.74 × 10^–4^
9835.6936	3.32 × 10^–35^	300243.872	290408.1788	2.72 × 10^–3^	2.72 × 10^–4^
9836.507	3.30 × 10^–35^	300271.319	290434.8118	2.70 × 10^–3^	2.70 × 10^–4^
9837.3436	3.29 × 10^–35^	300297.599	290460.255	2.69 × 10^–3^	2.69 × 10^–4^
9837.344	3.29 × 10^–35^	300297.598	290460.2544	2.69 × 10^–3^	2.69 × 10^–4^
9837.7536	6.33 × 10^–36^	301417.538	291579.7848	5.18 × 10^–4^	5.18 × 10^–5^

It appears that C_60_H^+^ has the right rotational structure for detection using the rotational comb method reinforced by the spectral match filtering. Figure 7 shows a J comb with tooth separation of about 167 MHz. Each tooth contains the K structure in its regular part as a finer comb with tooth separation of about 7.5 kHz. Thus, the rotational comb for each J+1 → J transition has teeth as a series of finer K combs.

**Figure 7:**
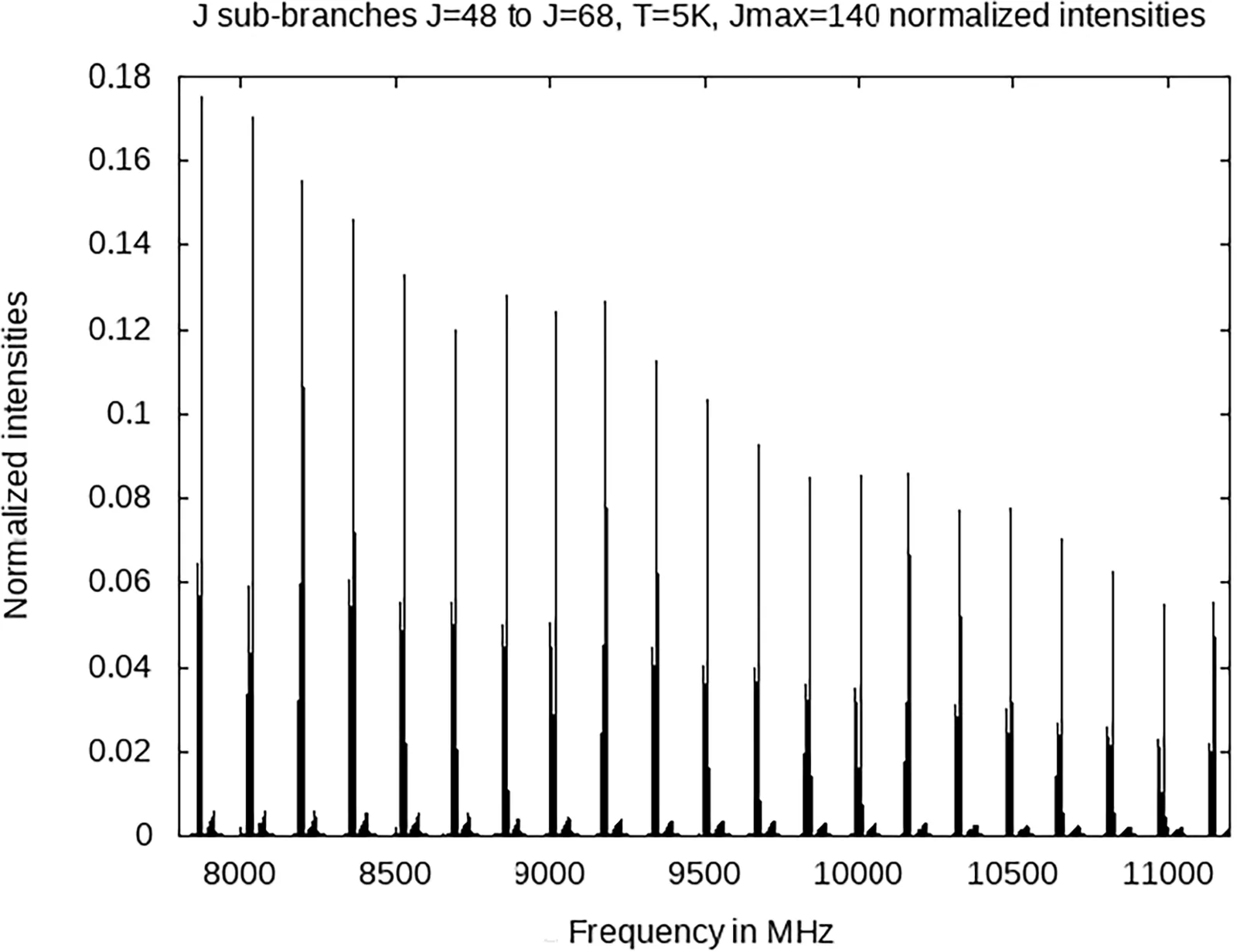
A series of J sub-branches at *T* = 5 K. These sub-branches lie in the X band of the Green Bank Radio Telescope. Each sub-branch contains a dense K structure. The separation between the J sub-branches is (B+C), which is about 164 MHz. The K structure in the J = 60 sub-branch is shown in Figure 8.

To detect C_60_H^+^ in the TMC-1 molecular cloud core at T = 5 K, the Green Bank Telescope (GBT) could be used in the X frequency band (8.0–11.6 GHz) using the VEGAS astronomical spectrometer that has sensitivity down to 0.7 kHz.

**Figure 8:**
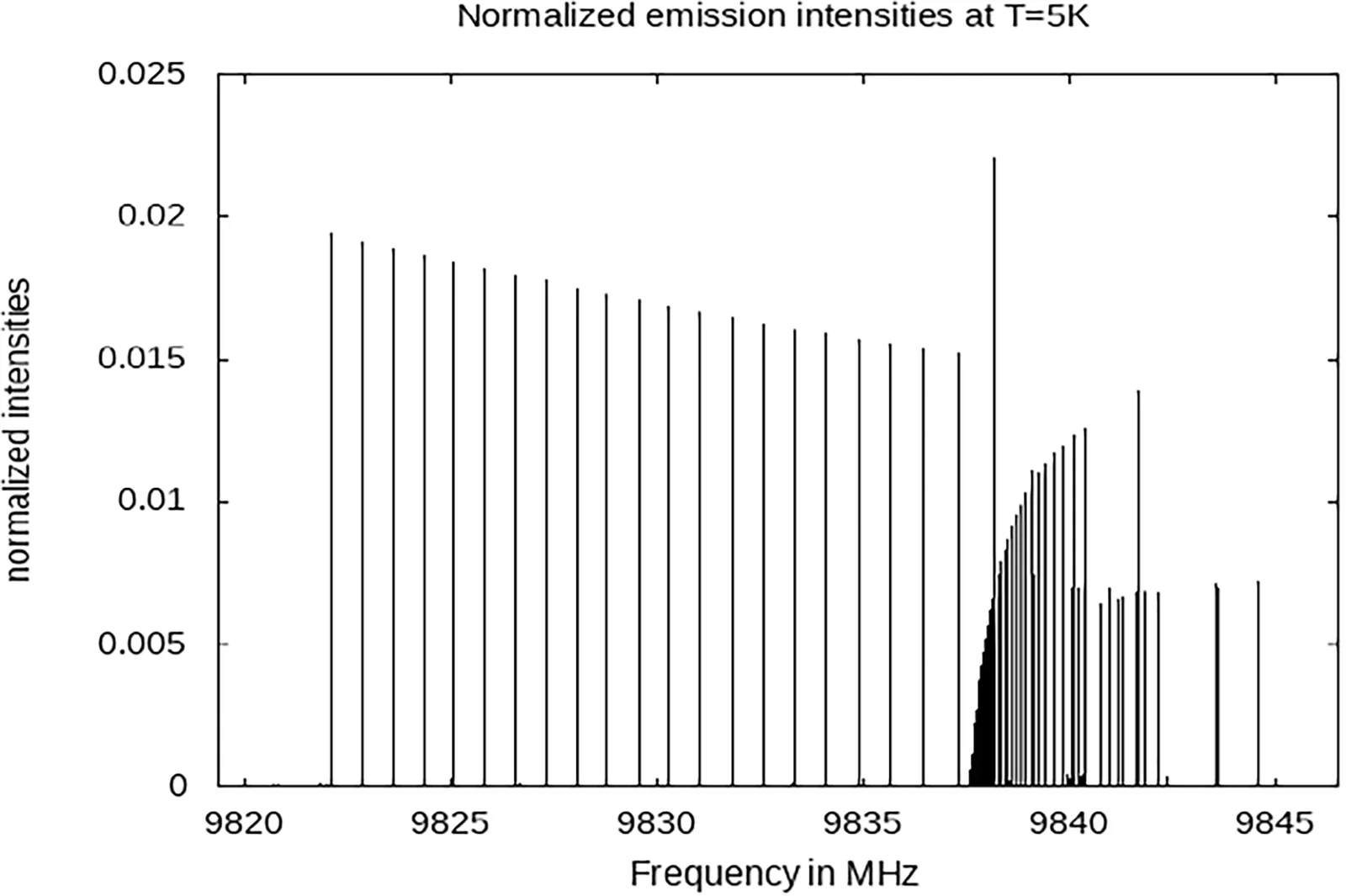
Detail of the emission spectrum of the J = 60 sub-branch for *T* = 5 K.

## Conclusions

We present predicted pure rotational spectra for protonated C_60_ at different temperatures. Rather than being a near-symmetric top, C_60_H^+^ is an unusual case of being near-spherical, which gives the species its peculiar rotational spectrum. Although rotational constants in the molecule-fixed frame were carefully derived from DFT-optimized geometries using harmonic and anharmonic approaches, line positions are not sufficiently accurate to allow for a direct search of astronomical spectra. Nonetheless, we believe that the present results provide new insights into the rotational structure of nearly symmetric polar fullerene derivatives and may guide future laboratory and astronomical studies.
